# Supplementary material for: The genome sequence of a tachinid fly, Linnaemya vulpina (Fallén, 1810)
Source: Wellcome Open Res. 2024 Nov 4;9:643. [Version 1] doi: 10.12688/wellcomeopenres.23296.1 (PMC11724206; doi:10.12688/wellcomeopenres.23296.1)
Supplement: Peer review history

# Reviewer report: Reviewer response for version 1

**DOI:** 10.21956/wellcomeopenres.25678.r113229

**Published:** 2025-01-10

**Authors:** Henrique Antoniolli

**Affiliations:** 1 Universidade Federal do Rio Grande do Sul (UFRGS), Porto Alegre, Brazil

The present study provides a highly contiguous and annotated genome assembly for a tachinid fly. Introduction is clear and concise; methods and bioinformatic tools are well described; and results are thoroughly described. I recommend indexing the paper.

**Minor comment: **The mitochondrial genome should also be annotated and deposited in public repositories.

Are sufficient details of methods and materials provided to allow replication by others?

Yes

Is the rationale for creating the dataset(s) clearly described?

Yes

Are the datasets clearly presented in a useable and accessible format?

Yes

Are the protocols appropriate and is the work technically sound?

Yes

Reviewer Expertise:

Genomics, evolution

I confirm that I have read this submission and believe that I have an appropriate level of expertise to confirm that it is of an acceptable scientific standard.

# Reviewer report: Reviewer response for version 1

**DOI:** 10.21956/wellcomeopenres.25678.r112249

**Published:** 2024-12-19

**Authors:** Kyle M Benowitz

**Affiliations:** 1 Arizona State University, Mesa, USA

This manuscript presents a high-quality genome of a fly species using a reliable assembly and annotation pipeline. The methods and results are standard and easy to follow, and I have no significant concerns. I'm happy to see this excellent resources added to the Tree of Life project.

 While I think ToL is a worthy goal in itself, I'd prefer to have seen a more direct statement about the kinds of questions that could be addressed with this genome. The authors state "This will aid research on taxonomy and biology of the species and the phylogeny of the family Tachinidae." This is of course true, but vague - what are the interesting biological questions that this species in particular can be studied for? I always think these genome reports gain value if authors can clearly state those questions.

 Minor comments:

 1. Is there a reason why the BUSCO scores in Figure 2 differ from those in Table 2 and in the text?

 2. Also, why wasn't BUSCO performed on the proteome to assess the completeness of the annotation?

Are sufficient details of methods and materials provided to allow replication by others?

Yes

Is the rationale for creating the dataset(s) clearly described?

Partly

Are the datasets clearly presented in a useable and accessible format?

Yes

Are the protocols appropriate and is the work technically sound?

Yes

Reviewer Expertise:

Evolutionary genomics, insects

I confirm that I have read this submission and believe that I have an appropriate level of expertise to confirm that it is of an acceptable scientific standard.
